# An evaluation of the quality of statistical design and analysis of published medical research: results from a systematic survey of general orthopaedic journals

**DOI:** 10.1186/1471-2288-12-60

**Published:** 2012-04-25

**Authors:** Nick R Parsons, Charlotte L Price, Richard Hiskens, Juul Achten, Matthew L Costa

**Affiliations:** 1 Warwick Medical School, University of Warwick, Coventry, CV2 2DX, UK; 2 Public Health, Epidemiology and Biostatistics Group, University of Birmingham, Birmingham, B15 2TT, Uk; 3 University Hospitals Coventry and Warwickshire NHS Trust, Coventry, UK

## Abstract

**Background:**

The application of statistics in reported research in trauma and orthopaedic surgery has become ever more important and complex. Despite the extensive use of statistical analysis, it is still a subject which is often not conceptually well understood, resulting in clear methodological flaws and inadequate reporting in many papers.

**Methods:**

A detailed statistical survey sampled 100 representative orthopaedic papers using a validated questionnaire that assessed the quality of the trial design and statistical analysis methods.

**Results:**

The survey found evidence of failings in study design, statistical methodology and presentation of the results. Overall, in 17% (95% confidence interval; 10–26%) of the studies investigated the conclusions were not clearly justified by the results, in 39% (30–49%) of studies a different analysis should have been undertaken and in 17% (10–26%) a different analysis could have made a difference to the overall conclusions.

**Conclusion:**

It is only by an improved dialogue between statistician, clinician, reviewer and journal editor that the failings in design methodology and analysis highlighted by this survey can be addressed.

## Background

Statistics is an essential component of medical research from design initiation to project reporting, and it influences all aspects of the research process from data collection and management to analysis and interpretation. The application of statistics to medical sciences, and particularly in our area of interest, trauma and orthopaedic surgery, has become more widespread and complex. However, there is considerable evidence, both anecdotal and in the literature [1], of poor reporting and use of statistical methods in orthopaedics papers. Although our experience providing statistical support more widely in medicine leads us to suspect that similar opinions, about the quality of both design and statistical analysis, exists within many other medical disciplines. So our selection of general orthopaedic journals is not solely to highlight particularly bad practice in this discipline, as we suspect much of what we report here is generally applicable to research across all disciplines, and as such orthopaedic publications simply provide an exemplar of this larger population. In an attempt to quantify the extent of poor reporting and use of statistical methods, Parsons et al. [2] undertook a large survey of the orthopaedic literature to assess both the quality of reporting and the appropriate and correct use of statistical methods. The first part of this study found major deficiencies in reporting, with 59% (95% confidence interval; 56–62%) and 58% (56–60%) compliance with CONSORT [3] and STROBE [4] guidelines, and commented on differences between journals and paper types [2]. In the second part of the study, the quality of statistical analysis methods was assessed using a detailed questionnaire which was completed for a random sample of orthopaedics papers by two experienced statisticians. The results of this survey are discussed in detail here.

## Methods

A random sample of 100 papers from the general orthopaedic literature was obtained and included 27 randomized controlled trials (RCTs), 30 case–control (CC) studies, 16 longitudinal (L) studies and 27 cross-sectional (CS) studies. The sample was stratified by study type to ensure accurate representation of each of the four types of study and additional inclusion criteria were as follows:

(i) Published research papers from seven general orthopaedic journals [5] covering a range of impact factors [6]; *Journal of Bone and Joint Surgery* (American), *Clinical Orthopaedics and Related Research, Journal of Bone and Joint Surgery* (British), *Acta Orthopaedica, Archives of Orthopaedic * and *Trauma Surgery, International Orthopaedics* and *BMC Musculoskeletal Disorders*

(ii) Original research only – excluding trial protocols, reviews, meta-analyses, short reports, communications and letters

(iii) Published between 1^st^ January 2005 and 1^st^ March 2010 (study start date)

(iv) No more than one paper from any single research group

(v) Papers published by research groups based at our own institutes were excluded to avoid assessment bias

Full details of the search strategy and methods used to collect the sample are provided by Parsons et al. [2].

The statistical quality of each paper was assessed using a validated questionnaire [7], which was adapted to reflect the specific application to orthopaedic research [2]. After randomly numbering the papers from 1 to 100, each paper was read and independently assessed using the questionnaire by two experiences statisticians (NP and CP). Even numbered papers were read by NP and odd numbered papers were read by CP. The questionnaire was divided into two parts. Part one captured data describing the type of study, population under study, design, outcome measures and the methods of statistical analysis and the results of this were reported in Parsons et al. [2]. A random sample of 16 papers from the original 100, stratified by study type to ensure balance, was selected and read by both statisticians to assess the level of agreement between the two reviewers for individual items on part one of the questionnaire. Parsons et al. [2] reported kappa statistics in the range 0.76 to 1.00 with a mean of 0.96 suggesting good agreement between the reviewers for this more objective part of the survey. The second part of the questionnaire required generally more subjective assessments concerning the presentation of data and the quality and appropriateness of the statistical methods used (see Additional file 1 for questionnaire details). The results of this part are reported in detail here. The survey allowed a detailed investigation of issues such as the description of the sample size calculation, missing data, the use of blinding in trials, the experimental unit, multiple testing and presentation of results.

The *correctness, robustness, efficiency * and *relevance *[7] of the statistical methods reported in the sample papers were assessed using a yes or no assignment for each characteristic. *Correctness* refers to whether the statistical method was appropriate. For instance, it is not correct to use an unpaired *t*-test to compare an outcome from baseline to the trial endpoint for a single group of patients. Many statistical methods rely on a number of assumptions (e.g. normality, independence etc.); if those assumptions are incorrect, the selected method can produce misleading results. In this context we would describe the selected methods as lacking *robustness*. A statistical method was rated as *inefficient* if, for example, a nonparametric rather than a parametric method was used for an analysis where data conformed to a known distribution (e.g. using a Mann–Whitney test, rather than a *t*-test). Finally, an analysis was regarded as *relevant* if it answered the question posed in the study. For instance, a principal components analysis may be correct and efficient for summarising a multivariate dataset, but may have no bearing on the stated aim of a paper.

The majority of the survey items were objective assessments of quality, e.g. an incorrect method of analysis was used, with a small number of more subjective items, e.g. could a different analysis make a difference to the conclusions?

## Results

The outcomes of part two of the statistical questionnaire are summarized in the following three subsections covering study design, statistical methods and the presentation of results.

### Study design

A number of key themes emerged from the analysis of the questionnaire data. Foremost amongst these were the description of the study design, identification of the experimental unit, details of the sample size calculation, the handling of missing data and blinding for subjective measures. These topics are discussed individually below.

(i) Experimental unit

The experimental unit is a physical object which can be assigned to a treatment or intervention. In orthopaedics research, it is often an individual patient. However, other possibilities include things such as a surgeon, a hip or a knee. The experimental unit is the unit of statistical analysis and, for simple study designs, it is synonymous with the data values, i.e. there is a single outcome measure for each experimental unit. For more complex designs, such as repeated measures, there may be many data values for each experimental unit. Failure to correctly identify the experimental unit is a common error in medical research, and often leads to incorrect inferences from a study [1,8].

The experimental unit was not identified correctly in 23% (15–33%; 95% confidence interval based on normal approximation to binomial) of the sampled studies. Of the 77 papers that correctly identified the experimental unit, 86% (75–92%) correctly summarised the data by patient. By far the most common reason for incorrect identification of the experimental unit was confusion between limbs and individual patients when analysing and reporting results. For example, one paper reported data for 100 patients but summarised outcomes for 120 feet, whereas another reported patient pain scores after surgery for both left and right ankles on some patients and single ankles for other patients. Failure to identify the correct experimental unit can lead to ‘dependencies’ in data. For example, outcome measures made on left and right hips for the same patient will be correlated, but outcome measures between individual hips from two different patients will be uncorrelated. Only one paper, where data were available from one or both legs for patients, identified this as an important issue and the authors decided to use what we would regard as an inappropriate strategy by taking the mean of the two values as the outcome for bilateral patients. Almost all of the statistical analyses reported in these studies (e.g. t-tests, ANOVA, regression) are based on an assumption that outcome data (formally the residuals) are uncorrelated; if this is not the case then the reported inferences are unlikely to be valid.

(ii) Sample size

The size of the sample used in a study, i.e. the number of experimental units (usually patients), largely determines the precision of estimates of study population characteristics such as means and variances. That is, the number of patients in the study determines how confidently we can draw inferences from the results of that study and use them to inform decisions about the broader population of patients with that particular condition or problem. In clinical trials, a pre-study power analysis is usually used to estimate the sample size [9], although methods are available for many other study types [10]. It is particularly important for RCTs, where specific null hypotheses are tested, that a clear description of the methodology and rationale for choosing a sample size is given. For example, the outcome is assumed to be normally distributed, treatment group differences will be assessed using a *t*-test, the power to detect a defined clinically meaningful difference is set to at least 80% and the type I error rate, or significance level, is set to 5%.

The sample size was not justified in the Methods section for 19% (7–39%) of the 27 papers describing RCTs. A specific calculation, with sufficient details to allow the reader to judge the validity, was not given for 30% (14–50%) of RCTs. These studies often simply described the sample size in vague terms, for instance *"…based on* a priori *sample size estimation, a total of 26 patients were recruited…"*. For 3 papers reporting RCTs, the validity of the sample size calculation was questionable, for 3 papers there was a lack of clearly stated assumptions and in 2 papers the calculation was simply not credible. For example, one paper gave sparse details about the population variance, minimum clinically important difference and required power which resulted in a recruitment target of 27 patients for a two arm trial. For purely practical reasons one would always want an even number of patients in a two arm trial. In another paper, 400 patients were recruited to a study, based on a vague description about how this number was arrived at, and exactly 200 patients were randomly allocated to each of two treatment groups. A cynical reader might question the likelihood of such an exact split of patients between treatment groups; there is only a 1 in 25 chance of an exact split for a simple 1 to 1 randomization. However, this might simply be a case of poor reporting, where in reality blocking or minimization were used to equalise numbers in the treatment arms, thus giving more credence to the description of the design. For the 73 observational studies, only 34% (24–46%) justified the sample size, that is there was some discussion in the paper on how the sample size was arrived at; this was often minimal, for instance a simple statement that the number of patients required to answer the research question was the number of patients who were available at the time of study, or those who accepted an invitation to participate (e.g. *"…all patients were invited to join the study…"*).

(iii) Missing data

Missing data are observations that were intended to be made but were not made [11]; the data may be missing for unexpected reasons (e.g. patient withdrawal from a study), or intentionally omitted or not collected. It is important to carefully document why data are missing in the study design when reporting. If data can be considered to be missing at random, then valid inferences can still be made. However, if values are missing systematically, then it is more dangerous to draw conclusions from that study. For example, if in a clinical trial comparing different types of hip replacement all of the missing data occurs in one particular arm of the trial, the remaining data is unlikely to be representative of the overall result in that group of patients; the missing data may be because those patients went to another hospital for their revision surgery.

Data were missing, either for a complete unit or a single observation, in 34% (25–44%) of the papers, of these 34 papers only 62% (44–77%) documented and explained the reasons for this. An audit of the data reported in each paper allowed the statistical assessors to identify 13 papers (13% of the total sample) where data were missing with no explanation. Data missingness was generally inferred from the numbers reported in the results being less than those reported in the methods, with no explanation or reason offered by the authors of the study. In the 34 papers reporting missing data, 28 based the analysis on complete cases, 2 imputed missing data and for the remaining 4 papers it was unclear as to what methodology was used.

(iv) Subjective assessments and blinding

Many orthopaedic studies report subjective assessments, such as a pain or a functional score after surgery or a radiological assessment of the quality of a scan. To reduce the risk of bias for these kinds of assessments it is desirable, where possible, to ‘blind’ the assessor to the treatment groups to which the patient was allocated.

Subjective assessments were undertaken in 16 of the 27 RCTs (59%; 95% CI 39–77%) and in 6 of these studies (38%; 95% CI 16–64%), the assessments were not done blind and no explanation was given as to why this was not possible.

### Statistical methods

Statistical methods should always be fully described in the methods section of a paper and only the statistics described in the methods should be reported in the results section. In 20% (13–29%) of the papers in our survey, statistical methods not previously stated in the methods section were reported in the results section [2]. In addition to the poor reporting of the methods used, a number of specific issues were identified.

(i) Analysis methods

The most commonly reported statistical methods were chi-squared (χ^2^) and Fisher’s exact tests (47%; 95% CI 37–57%), t-tests (45%; 95% CI 35–55%), regression analysis (33%; 95% CI 24–43%) and Mann–Whitney tests (28%; 95% CI 20–38%). The selection of an appropriate method of analysis is crucial to making correct inferences from study data.

In 52% (32–71%) of papers where a Mann–Whitney, Wilcoxon rank sum or Wilcoxon signed rank test was used, the analysis was considered to be inefficient and the reported analysis was only considered to be correct 70% (50–86%) of the time. The *t*-test was used inappropriately, with a lack of robustness, in 26% (14–41%) of papers and in an equivalent proportion of papers (26%; 95% CI 14–41%) it was reported in such a way as to be irrelevant to the stated aims of the paper. This lack of relevance was, on occasion, due to method selection such as the choice between a parametric and a nonparametric test, but more often was simply a result of poor reporting and lack of clarity in the description. Many papers reported a list of the statistical tools used in the analysis, but in the results gave only short statements such as *“A was better than B (p = 0.03)”* with no details as to which test was used to obtain the p-value; so-called ‘orphan’ p-values [12]. It was therefore impossible to assess whether the correct test was used for the relevant comparison.

Seven papers (7%; 95% CI 3–14%) reported clear methodological errors in the analysis. Two papers wrongly used the Wilcoxon signed-rank test to compare independent samples and another paper used an independent samples *t*-test where a paired test should have been used. One paper committed the reverse error of using a paired *t*-test to compare cases and controls in an unpaired case–control study and another paper used a *t*-test to compare differences in proportions rather than, for instance, a χ^2^ test. Another study calculated the arithmetic mean of a number of percentages, all based on different denominator populations. And finally, one study outlined reasons for conducting a non-parametric analysis in the methods only to later report an analysis of covariance, a parametric method of analysis based on assumptions of normality.

(ii) Parametric versus non-parametric tests

Parametric statistical tests assume that data come from a probability distribution with a known form. That is, the data from the study can be described by a known mathematical model; the most widely used being the normal distribution. Such tests make inferences about the parameters of the distribution based on estimates obtained from the data. For example, the arithmetic mean and variance are parameters of the normal distribution measuring location and spread respectively. Non-parametric tests are often used in place of parametric tests when the assumptions necessary for the parametric method do not hold; for instance the data might be more variable or more skewed than expected. However, if the assumptions are (approximately) correct, parametric methods should be used in preference to non-parametric methods as they provide more accurate and precise estimates, and greater statistical power [13].

Many of the papers in this survey showed no clear understanding of the distinction between these types of tests, evidenced by reporting that made no statistical sense: e.g. *"…continuous variables were determined to be parametric using Kolmogorov-Smirnov tests…"*, *"…the t-test was used for parametric variances…"*, *"…non-parametric statistics were used to compare outcome measures between groups (one way ANOVA)…"* and *"…Student's t-test and the Mann–Whitney test were used to analyse continuous data with and without normal distribution…"*. Continuous variables may be assumed to be approximately normal in an analysis, but it makes no sense to describe variables or variances as parametric. It is also incorrect to label an analysis of variance (ANOVA) as non-parametric. In at least 5 papers (5%; 95% CI 2–12%), the authors opted to use non-parametric statistical methods, but then summarised data in tables and figures using means and standard deviations, the parameters of the normal distribution, rather than correctly using medians and ranges or inter-quartile ranges.

The survey showed that 52% (42–62%) of papers used non-parametric tests inefficiently; that is they reported the results of non-parametric tests for outcomes that evidence from the paper suggested were approximately normal. Three papers (3%; 95% CI 0–9%) compared the lengths of time to an outcome event between groups by using the non-parametric Mann–Whitney (M-W) test based on converting the times to ranks. By doing this, much of the information about real differences between individual records is lost; for example outcomes of 1 day, 2 days and 100 days become 1, 2 and 3 when converted to ranks. Although times are often positively skewed, they are usually approximately normally distributed after logarithmic transformation [14]. A more efficient analysis can therefore usually be achieved by using a *t*-test on log-transformed times rather than applying a M-W test to untransformed data. This is not to say that non-parametric tests should never be used, but that for many variable types (e.g. times, areas, volumes, ratios or percentages) there are simple and well-known transformations that can be used to force the data to conform more closely to the assumptions required for parametric analysis, such as normality or equality of variances between treatment groups.

(iii) Multiple comparisons

Problems of multiple comparisons, or multiple testing, occur when considering the outcomes of more than one statistical inference simultaneously. In the context of this survey, it is best illustrated by considering a number of reported statistical tests for one study all reporting evidence for significance at the 5% level. By definition, if one undertakes 20 hypothesis tests on data where we know that there is no true difference, we will expect to see one significant result at the 5% level by chance alone. Therefore, if we undertake multiple tests, we require a stronger level of evidence to compensate for this. For example, the Bonferroni correction preserves the ‘familywise error rate’ (α), or the probability of making one or more false discoveries, by requiring that each of n tests should be conducted at the α/n level of significance, i.e. it adjusts the significance level to account for multiple comparisons [15].

The questionnaire recorded the number of hypotheses tested in each paper, based on an approximate count of the number of p-values reported. Three papers did not report p-values, 31 papers (31%; 95% CI 22–41%) reported less than 5 p-values, 36 papers (36%; 95% CI 27–46%) reported between 5 and 20 p-values and 30 papers (30%; 95% CI 21–40%) reported more than 20 p-values. Issues of the relevance and the need for formal adjustment for multiple comparisons will clearly be very problem specific [16]. Whilst most statisticians would concede that the formal adjustment of p-values to account for multiple comparisons may not necessarily be required when reporting a small number of hypothesis tests, if reporting more than 20 p-values from separate analyses, some discussion of the rationale and need for so many statistical tests should be provided and formal adjustment for multiple-comparison considered. In an extreme case, one paper reported a total of 156 p-values without considering the effect of this on inferences from the study. A Bonferroni correction to the significance level would have resulted in at least 21 of the 35 reported significant p-values in this study to be regarded as no longer significant. Where some adjustment was made for multiple comparisons (7 papers), the Bonferroni correction was the most common method (5 papers). One other paper used Tukey’s Honestly Significant Difference (HSD) test and another set the significance level to 1% (rather than 5%) in an ad-hoc manner to account for undertaking 10 tests.

### Presentation of results

The clear and concise presentation of results, be it the labelling of tables and graphs or the terminology used to describe a method of analysis or a p-value, is an important component of all research papers. The statistical assessment of the study papers identified two important presentational issues.

(i) Graphs and tables

The statistical assessors were asked to comment on the quality of the data presentation in the papers which included tables and graphs. Graphs and tables were clearly titled in only 29% (21–39%) of papers. For instance, typical examples of uninformative labels included *“Table I: Details of Study”* and *“Table II: Surgical Information”*. Furthermore, only 43% of graphs and tables were considered to be clearly labelled. In particular, a number of the papers included tables with data in parentheses without further explanation. The reader was then left to decide whether the numbers indicated, for example, 95% confidence intervals, inter-quartile ranges (IQRs) or ranges. Some tables also included p-values with no indication of the statistical test used. The description of graphical displays was occasionally confusing. One paper stated that the bars of a box-and-whisker plot represented the maximum and minimum values in a dataset, when there were clearly points outside the bars. By convention, the bars represent 1.5 times the inter-quartile range, with points outside the bars identified as ‘outliers’. Interestingly, another paper claimed that the boxes showed standard deviations, rather than the correct IQR, so there is clearly a wider misunderstanding of these figures.

Raw data for individual patients (or experimental units) were displayed graphically or in tables in only 9% (4–17%) of papers. Raw data, as opposed to means, medians or other statistics, always provide the simplest and clearest summary of a study, and direct access to the data for the interested reader. Although we accept that there may be practical reasons why authors would not want to present such data, it is disappointing that such a small proportion of investigators decided to do so.

(ii) Terminology

The lack of appropriate statistical review, either prior to submission or at the review stage, was apparent in the catalogue of simple statistical reporting errors found in these papers. For instance, methods were reported that, to our knowledge, do not exist: e.g. *"multiple variance analysis"* or the *“least squares difference"* post-hoc test after ANOVA. Presumably the latter refers to a least significant difference test, but the former is ambiguous. Another class of reporting error were those that simply made no statistical sense in the context they were reported: e.g. *"…there was no difference in the incidence among the corresponding groups (chi-squared test, p = 0.05)…"*, and *"…there were no significant differences in the mean T-score or Z-score between the patients and controls…"*. The former remark was made in the context of rejecting the null hypothesis at the 5% level for significance and the latter presumably implied that mean t-statistics and z-scores were compared between groups, which makes no statistical sense. The inadequate or poor reporting of p-values was also widespread, and typical errors included *"p < 0.000009"*, *“p < 0.134”* and, more generally, the use of *“p = NS”* or *“p < 0.05”*. P-values should generally be quoted to no more than 3 decimal places, and be exact (as opposed to an inequality e.g. p < 0.05), unless very small when p < 0.001 is acceptable.

## Discussion

A number of key issues have emerged from our survey of 100 papers investigating the quality of statistical analysis and design in trauma and orthopaedic research. These points are summarised below with recommendations for improvement.

### Experimental unit

It is important that authors clearly identify the experimental unit when reporting. This was a source of confusion for 23% (95% CI 15–33%) of the papers in our survey and reflects a fundamental lack of understanding. If no attempt is made to modify the analysis to account for data dependencies, researchers should at least state that they are making an assumption of (approximate) independence between multiple observations for the same unit (e.g. functional outcomes from the left and right side for the same individual after a bilateral procedure). This then at least allows the reader to decide whether the assumption is reasonable in the context of the study.

### Sample size

Where specific hypotheses are being tested, for instance in an RCT, the sample size should be discussed, and usually a power calculation reported with sufficient details to allow one to verify the reported sample size. In this survey, 30% (14–50%) of the RCTs gave no such calculation and 19% (7–39%) of them provided no justification for the sample size in the methods section. A clear description of the methodology used for sample size determination (e.g. power level, significance) and the design used (e.g. randomization) is critical for judging the quality of research. However, in studies where a sample size calculation may not be relevant, for example if the research is exploratory or researchers have access to a limited number of participants, authors should provide an open discussion of this in the methods section.

### Missing data

The lack of a clear explanation offered by a number of the papers in this survey for missing data goes hand-in-hand with the poor reporting of patient numbers. Parsons et al. [2] showed that only 57% of these papers stated exact numbers of patients in both the methods and results sections. RCTs should report a (CONSORT-style [3]) flowchart documenting exactly what happened to all of the participants in the trial, and all studies should state how and why any patient data was missing or excluded from the analysis. Furthermore, all studies should state the size of sample used to estimate parameters, as a way of explicitly stating whether all or only partial information was available for inference.

### Blinding

It is important for the credibility of reported research to take all practical steps to remove potential sources of bias from a study. Blinding an assessor to the treatment allocation in RCTs is a simple method to achieve this. We expect that when subjective scores are used then blinding is necessary, or if blinding is not possible some explanation should be offered as to why it was not possible or practical.

### Analysis methods

This survey has highlighted the common use of inefficient or irrelevant statistical methods, with 7 papers reporting clear methodological errors. Not only does this suggest that many of the studies reported in these papers have had little or no expert statistical input, it is clear that many of the papers have not undergone adequate statistical review prior to publication. The lack of clear association between the description of the statistical methods and the reporting of the outcome (e.g. p-value) was widespread. However, this kind of issue could be easily corrected by obtaining appropriate expert statistical review prior to submission. If a reviewer notes a statistical error, or does not understand a statistical analysis plan, they should recommend that an expert opinion is sought during the review process after submission to the journal.

### Parametric versus non-parametric tests

Non-parametric tests were used widely in the studies in this survey in a manner that suggested a lack of understanding as to when they are appropriate. For instance when selecting between a (parametric) *t*-test or a (non-parametric) Mann–Whitney test, the latter test should only be used for outcomes that are not *approximately* normally distributed and should be reported with medians and ranges (or interquartile ranges), not means and standard deviations [13]. However, where a natural transformation is available to make an outcome ‘more normal’, undertaking the analysis on the transformed scale using normal test statistics is usually a more efficient and powerful option than the non-parametric alternative. The widespread misuse of non-parametric tests in this survey suggests that this issue is not widely appreciated.

### Multiple comparisons

Carrying out multiple hypothesis tests was a common practice in many of the papers reviewed, with 30% (21–40%) of the papers reporting over 20 p-values and one study reporting a massive 156. Whilst we accept that the details of statistical methods to correct for multiple comparisons may not be common knowledge in the orthopaedic research community, and the circumstances when it is appropriate to adjust for multiple testing remain contentious amongst both statistical and clinical communities [16], we would expect most researchers to have some appreciation that carrying out large numbers of hypothesis tests and reporting significance at the standard 5% level is bad practice. We would advise that often the best way of dealing with this issue is to insist that a clear description and justification of all tests of significance that have been performed be included; this process of questioning will generally lead to a marked reduction in the number of tests reported. *Graphs and tables:* The presentation of results was a particular weak point in the papers included in this survey. All graphs and tables should be clearly labelled and sufficiently detailed to allow at least some inference to be made in isolation from the rest of the paper. Authors should include a clear title so that readers can quickly understand the information on display without reference to the main body of the text. With the increasing availability of software for producing sophisticated plots, it is tempting for authors to indulge in novel ways to present results. However, this is often one area where clear and simple tends to be best.

Although not formally one of the items in the questionnaire, we noted that 5 of the 27 RCTs (19%; 95% CI 7–39%) tested for differences between baseline characteristics (e.g. age, gender ratio, BMI etc.) after recruitment and randomization of patients to treatment arms of a trial. Since the randomization process produces treatment groups that are random samples from the *same population*, a null hypothesis of no difference between two populations must, by definition, be true. As such, any significant difference observed between groups must have arisen by chance; i.e. it is a type I error. Despite the widespread appreciation of this argument within the statistics community, this is still a widely reported error in many medical disciplines that, with adequate statistical input during the course of a study and at review, could be avoided.

## Conclusion

The opinions expressed here are the result of independent assessments made by two statisticians using a sample of 100 representative orthopaedic papers and, as such, are limited by the experience and prejudices of the assessors and the size and nature of the survey. However, the carefully designed sampling strategy and the random selection methods ensured that the papers surveyed were indeed representative of the target literature [2]. Furthermore, the fact that many of the issues highlighted in this paper are familiar topics to those providing statistical reviews of medical research [17], suggests that the views expressed here are likely to be widely held within this community. For those who are unfamiliar with good practice in research, others have provided guidance in the use of statistics in the orthopaedic setting [1,18] and also specifically in the design of RCTs [19,20]. More generally, the series of short articles on the use of statistics for medical researchers published in the *British Medical Journal*[21] provide a rich resource of information on good statistical practice. Although our focus here has been on research published in general orthopaedic journals, the nature and extent of the issues raised here are clearly not exclusive to this discipline and as such we expect that the issues raised in the discussion and our recommendations for improvement to be applicable across all medical disciplines. To the non-statistically trained reader, many of the criticisms reported here may seem niggling and unimportant relative to the clinical details of a study. However, it is troubling to report that the statistical assessors in this survey thought that in 17% (10–26%) of the studies, the conclusions were not clearly justified by the results. For 39% (30–49%) of studies a different analysis should have been undertaken and for 17% (10–26%) of them, a different analysis could have made a difference to the conclusions. The results of this survey present challenges for us all, whether statistician, clinician, reviewer or journal editor, and it is only by greater dialogue between us all that these important issues can be addressed.

## Competing interests

The authors declare that they have no competing interests.

## Authors’ contributions

NP, JA, MC, RH and CP designed the study and extracted the data. RH identified the papers for inclusion in the study. NP and CP, reviewed the papers, extracted the data, conducted the analyses and created the first draft of the manuscript. All authors participated in editing the manuscript and approved final manuscript for publication.

## Pre-publication history

The pre-publication history for this paper can be accessed here:

http://www.biomedcentral.com/1471-2288/12/60/prepub

## Supplementary Material

Additional file 1 Statistical Questionnaire. Click here for file
