# Supplementary material for: An evaluation of the quality of statistical design and analysis of published medical research: results from a systematic survey of general orthopaedic journals
Source: BMC Med Res Methodol. 2012 Apr 25;12:60. doi: 10.1186/1471-2288-12-60 (PMC3476984; doi:10.1186/1471-2288-12-60)
Supplement: Additional file 1 — Statistical Questionnaire. [file 1471-2288-12-60-S1.doc]

**An Evaluation of the Quality of Statistical Design and Analysis of Published Medical Research: Results from a Systematic Survey of General Orthopaedic Journals**

MS: 1351510335627932

We thank the reviewers for their work and positive comments regarding this manuscript. The new version of the manuscript takes into account all the comments of the reviewers. A detailed list of the responses to individual comments is provided below.

**Reviewer 1: Nikolaos Pandis**

Reviewer's report: I think this is an easy to follow good descriptive paper regarding the statistical status of the specialty of interest. The only comment I would like to make is that I believe the use of a few tables summarizing the results would be helpful for the reader

Level of interest: An article of importance in its field. Quality of written English: Acceptable. Statistical review: No, the manuscript does not need to be seen by a statistician. Declaration of competing interests: I declare that I have no competing interests

*Authors Response: We considered carefully how we might report the results of the survey when preparing the manuscript and decided on a narrative approach rather than a more formal approach using tables and for instance a Forest plot for the main results (as suggested by Reviewer 3). We feel that this approach is the best way to review the broad range of issues identified in a way that is accessible to the non-statistician, but we are happy to accept advice on this issue if the Reviewer/Editor feel strongly about it.*

**Reviewer 2: Bengt I Eriksson**

Reviewer's report: This study is of high importance and could have impact on researchers. Methodology is correct. Results are presented in a clear way. Discussion is covering the subject and the discussing the results of the study in good way. Conclusions are well supported by the results of the study

Minor comments: The four categories of studies (RCT, CC, L, CS) are very different, hence it would have been very interesting also to see separate analyses of these different categories of studies.

It would also be interesting to compare different journals in a simple way by presenting a kind of 'range' of quality, i.e. identify a few key quality criteria and based on this present how many journals did have acceptable quality, i.e. were at the upper end of the quality scale and how many did not have acceptable quality regarding these key quality criteria.

Level of interest: An article of importance in its field. Quality of written English: Acceptable. Statistical review: No, the manuscript does not need to be seen by a statistician. Declaration of competing interests: I declare that I have no competing interests

*Authors Response: The reviewer is correct, the paper types are very different and as such the type and nature of the material reported will be very different. The most direct and easy way to obtain an assessment to compare quality and adherence to standards is to look at compliance to widely used standards of research reporting. To this end we determined compliance to CONSORT and STROBE guidelines for each paper in the survey; this information is available in the companion publication to this manuscript (Parsons N, Hiskens R, Price CL, Achten J, Costa ML.A systematic survey of the quality of research reporting in general orthopaedic journals. Journal of Bone and Joint Surgery (British) 2011; 93:1154-1159) which reports compliance by journal and paper type - exactly the information that the reviewer is requesting. We have emphasised this more clearly in the manuscript, at the end of the Introduction section.*

**Reviewer3: Erik Cobo**

Reviewer's report: This paper is well founded, easy to read and useful for readers. I made some suggestions so that authors may consider them in improving the paper.

Major suggestions:

1) Please, to further disseminate your message, consider concentrating in major problems and to avoid relying in minor deficiencies or on aspects that may be considered an error by you but not to any statistician. (Remember: “to avoid statisticians governing your committee, just put them in an even number”). For example, from my point of view, the paper cited as correct (page 7, line 6) is still wrong, since making the average will decline its dispersion and the data will no longer pertain to the same population with the same variance.

*Authors Response: We agree with the reviewer – taking the mean is not a good idea in this scenario. We cited this example not to suggest that it was correct, but rather this was the only paper that actually reported what they did and noted that this was an important issue – none of the other papers that reported data of this type made any explicit statement of how the analysis was modified to reflect this. We have modified this statement to indicate that we are not advocating this as the correct strategy – we are simply urging authors to make it clear what was actually done.*

As another example, multiplicity is a very sensible topic (see BMJ 1998;316:1236–8). From my point of view, it is almost straightforward in the Neyman-Pearson decision framework to identify the hypothesis set that leads to the same decision (i.e, give permission to market a drug) and need adjustment. But from the Fisher evidence point of view (most medical papers), several different scientific questions may be addressed with the same data. So, assessing if authors fall into multiplicity can be a very difficult and sensible question.

*Authors Response: Multiplicity is clearly a difficult topic for both clinicians and statisticians reporting research findings. We are not suggesting that modifications such Bonferroni corrections are used for all reported p-values. However, we do see this as an important issue that particularly non-statisticians should be much more aware of – in our experience it is not unusual to see papers reporting many 100 of p-values with little appreciation or understanding on the part of the authors that this might not be the best way to report the results of the analysis. The lack of understanding of these issues is particularly widespread in orthopaedics research, and as such we do feel it important to highlight this issue to this particular audience and as we say in the discussion “…we would expect most researchers to have some appreciation that carrying out large numbers of hypothesis tests and reporting significance at the standard 5% level is bad practice”. We have tempered our views on this issue and added appropriate references to help authors decide if this is an issue for their study, and to indicate that it is still a contentious subject amongst statisticians and made it clear that there should be a clear statement in all published work simply describing what tests of significance have been performed, and why.*

As a further example, I also prefer a parametric analysis after some transformation than a nonparametric one (pages 12 and 17), but only because it could provide a more readable effect size measure! I mean that this choice is, from my point of view, not a mistake, just free will. As a solution, please provide always references for your assessments. If possible, they should be on consensus documents such as reporting guidelines. To end this point, please consider concentrating your paper on main messages with solid documentation on agreement among statisticians and methodologists.

*Authors Response: We clearly did not set out our position on this issue in the manuscript. We would agree that often the choice of parametric versus non-parametric tests is a matter of choice. However, we would argue that for many papers we read as part of this survey non-parametric methods were used where evidence provided elsewhere in the paper indicated that the outcome measure was approximately normally distributed. In this setting, for instance a non-parametric Mann-Whitney test will be less powerful than a t-test. This is not widely appreciated amongst many non-statisticians – where often the message seems to be that a nonparametric test is preferable because it relies on fewer assumptions. The fact that by making this choice, for measures that are approximately normally distributed, we are diminishing our power to detect a difference between interventions (if it exists) is not widely appreciated. We have modified the text to convey this message more clearly and added appropriate references.*

2) There is a lack of statistical inference tools to generalize the results of your ‘representative’ sample to the represented population. For any reader it would be useful to know the sampling uncertainty in making inferences. Please, seriously consider adding confidence intervals for your reported proportions.

*Authors Response: Confidence intervals added as suggested.*

Minor suggestions:

1) To facilitate reading, please consider adding tables or, better, graphics. For example, a Forest Plot for the main results.

*Authors Response: See response to Reviewer 1.*

2) Please, consider if ‘orphan p value’ is easy to be measured and worth commenting on. My guess is that most of your papers don’t report ‘effect size’ measures to address clinical relevance.

*Authors Response: The term ‘orphan p value’ is not familiar to us – but we understand it to mean a p-value stated without a clear explanation of what test statistic was used to calculate it. This was not something that we specifically counted as part of the survey, but it was noted as an issue in the general feedback from the statistical assessors. We note this in the section on analysis methods and now add a reference to orphan p-values.*

3) In page 6, paragraph ‘experimental unit’ needs a reference for the statement ‘…is a common error…’.

*Authors Response: References have been added.*

4) Please, consider avoiding vague expressions such as ‘some cases’, ‘for a number of’ or ‘others’, such as in lines 3-5 of page 8.

*Authors Response: Where possible, changes have been made to the manuscript to tighten-up the text, as suggested by the reviewer. The nature of the survey was such that some of the statements reported in the manuscript are the result of comments made by the statistical reviews as issues emerged in the survey that were not part of the formal questionnaire, and therefore without a retrospective reassessment of the papers it is impossible to quantify frequencies exactly for some issues we highlight. We have attempted to make it clear in the manuscript where this is the case.*

5) You already mentioned the different role of ‘sample size rationale’ in confirmatory and exploratory studies, which is reflected in the different wording in Consort (‘pre-determined’) and Strobe (‘arrived at’). So, please clarify what you mean by ‘justified the sample size’ in line 16, page 8.

*Authors Response: We have clarified this in the text – as we state in the Discussion section a justification may simply be “…an open discussion in the methods section”. That is if a formal power analysis cannot be attempted, a discussion of constraints, practicalities and precedent.*

6) Please consider clarifying if ‘data missing’ in line 6, page 9 refers to a complete unit, or just to a single value.

*Authors Response: It refers to either or both of these options – this is now clarified in the text.*

7) Please, note that the Wilcoxon and the Mann Whitney tests are so closely related that some authors employ the name ‘Wilcoxon’ to refer to both paired and unrelated samples (page 11, line 2).

*Authors Response: We have modified the text a bit to clarify. What we referred to (Page 10) as Mann-Whitney or Wilcoxon signed rank tests were papers where MW, Wilcoxon rank sum or signed tests were reported; the former pair of tests we would regard as being equivalent. The two papers we report on Page 11 definitely used the term Wilcoxon signed rank tests to compare independent samples. It may be a case of a misnomer (i.e. they meant to say a Wilcoxon rank sum test) or a case of the incorrect use of a test – we cannot know from the papers.*

8) Please, specify on page 11, line 6, that the case-control study was not paired.

*Authors Response: Changed to unpaired case-control study as suggested.*

9) Please, clarify why the sentence on page 12, line 3 is so wrong.

*Authors Response: "…Student's t-test and the Mann-Whitney test were used to analyse continuous data with and without normal distribution…". We object to this type of statement on the basis that it suggests that there is some strict criterion that determines whether an outcome measure is or is not normal. This is an assumption that needs to be assessed carefully by the analyst. We often see reports of analyses from studies where for instance each outcome is assessed for normality using the significance of a Shapiro-Wilks test (that we know is to a large part dependent on the sample size!) as a strict decision rule for the type of test to be used. This statement is typical of analyses conducted in this manner, and propagates the view, that is widespread in orthopaedic research, that normality is a property of all outcomes that can be simply assessed and tested for. The reality is that the decision is more complex and depends on precedent, the type of outcome and many other issues.*

10) Please, consider removing ‘ethical’ in page 15, line 2. It should be understood that authors will delete any identification before publishing it. From my point of view, individual details belong to units, but results (with inference) belong to the population. What could be seen as unethical is keeping the anonymised data away from society.

*Authors Response: Agreed. This has been removed from the manuscript.*

Level of interest: An article of importance in its field. Quality of written English: Acceptable. Statistical review: Yes, and I have assessed the statistics in my report. Declaration of competing interests: 'I declare that I have no competing interests'

15^th^ March 2012

Nick Parsons (nick.parsons@warwick.ac.uk)

Charlotte Price (c.l.price@bham.ac.uk)

Richard Hiskens (rahiskens@doctors.org.uk)

Juul Achten (j.achten@warwick.ac.uk)

Matthew Costa (matthew.costa@warwick.ac.uk)
